# Expression profiling of migrated and invaded breast cancer cells predicts early metastatic relapse and reveals Krüppel-like factor 9 as a potential suppressor of invasive growth in breast cancer

**DOI:** 10.18632/oncoscience.10

**Published:** 2013-01-18

**Authors:** Ridha Limame, Ken Op de Beeck, Steven Van Laere, Lieselot Croes, Annemieke De Wilde, Luc Dirix, Guy Van Camp, Marc Peeters, Olivier De Wever, Filip Lardon, Patrick Pauwels

**Affiliations:** ^1^ Center for Oncological Research (CORE), University of Antwerp, Universiteitsplein 1, B-2610 Antwerp, Belgium; ^2^ Department of Medical Genetics, University of Antwerp and Antwerp University Hospital, Universiteitsplein 1, B-2610 Antwerp, Belgium; ^3^ Translational Cancer Research Unit (TCRU), GZA Hospitals Sint-Augustinus, Oosterveldlaan 24, B-2610 Wilrijk (Antwerp), Belgium; ^4^ Department of Oncology, KU Leuven, Herestraat 49, B-3000 Leuven, Belgium; ^5^ Laboratory of Pathology, Antwerp University Hospital, Wilrijkstraat 10, B-2650 Edegem (Antwerp), Belgium; ^6^ Department of Oncology, Antwerp University Hospital, Wilrijkstraat 10, B-2650 Edegem (Antwerp), Belgium; ^7^ Laboratory of Experimental Cancer Research, Department of Radiotherapy and Experimental Cancer Research, Ghent University Hospital, De Pintelaan 185, B-9000 Ghent, Belgium; ^8^ These authors equally contributed to this work.

**Keywords:** breast cancer, invasion, migration, KLF9, gene expression

## Abstract

Cell motility and invasion initiate metastasis. However, only a subpopulation of cancer cells within a tumor will ultimately become invasive. Due to this stochastic and transient nature, in an experimental setting, migrating and invading cells need to be isolated from the general population in order to study the gene expression profiles linked to these processes. This report describes microarray analysis on RNA derived from migrated or invaded subpopulations of triple negative breast cancer cells in a Transwell set-up, at two different time points during motility and invasion, pre-determined as “early” and “late” in real-time kinetic assessments. Invasion- and migration-related gene expression signatures were generated through comparison with non-invasive cells, remaining at the upper side of the Transwell membranes. Late-phase signatures of both invasion and migration indicated poor prognosis in a series of breast cancer data sets. Furthermore, evaluation of the genes constituting the prognostic invasion-related gene signature revealed Krüppel-like factor 9 (*KLF9*) as a putative suppressor of invasive growth in breast cancer. Next to loss in invasive vs non-invasive cell lines, *KLF9* also showed significantly lower expression levels in the “early” invasive cell population, in several public expression data sets and in clinical breast cancer samples when compared to normal tissue. Overexpression of EGFP-KLF9 fusion protein significantly altered morphology and blocked invasion and growth of MDA-MB-231 cells *in vitro*. In addition, *KLF9* expression correlated inversely with mitotic activity in clinical samples, indicating anti-proliferative effects.

## INTRODUCTION

Breast cancer remains the most frequently diagnosed malignancy and the primary cause of cancer-related death in women globally [1]. The heterogeneity of this disease, both inter- and intratumoral, contributes to its morbidity and underlines the necessity for personalized prognostics and therapeutics. Metastasis marks the final, systemic stage of advanced disease and results from a multistep process involving detachment of cells from the primary tumor, entry into and survival within the vasculature, arrest and exit into distant organ parenchyma, followed by formation of micrometastatic lesions, eventually developing into overt metastasis [2]. As disseminated disease accounts for > 90% of cancer mortality [3], there is an ongoing need for identification of relevant genes within this process.

Local invasive growth represents an initiating event predetermining metastatic spread and consists of a highly orchestrated interplay of transcriptional and signaling changes [1, 4, 5]. Paradoxically however, it has been established that only a small fraction of primary tumor cells become invasive and ultimately metastatic [2, 6]. Moreover, variability in the locoregional distribution of invasiveness within individual primary tumors tends to illustrate intratumoral heterogeneity [3, 6-8]. A significant proportion of cancer cell motility and invasion is propelled by chemotaxis, whereby cells move directionally along a gradient of soluble factors [9, 10]. Although modes of collective invasion have been recognized to play an important role in the biology of tumor progression, an important contribution to this process is added by invasive behavior generated on a single-cell basis, driven by epithelial-to-mesenchymal transition (EMT) [11].

**Figure 1:**
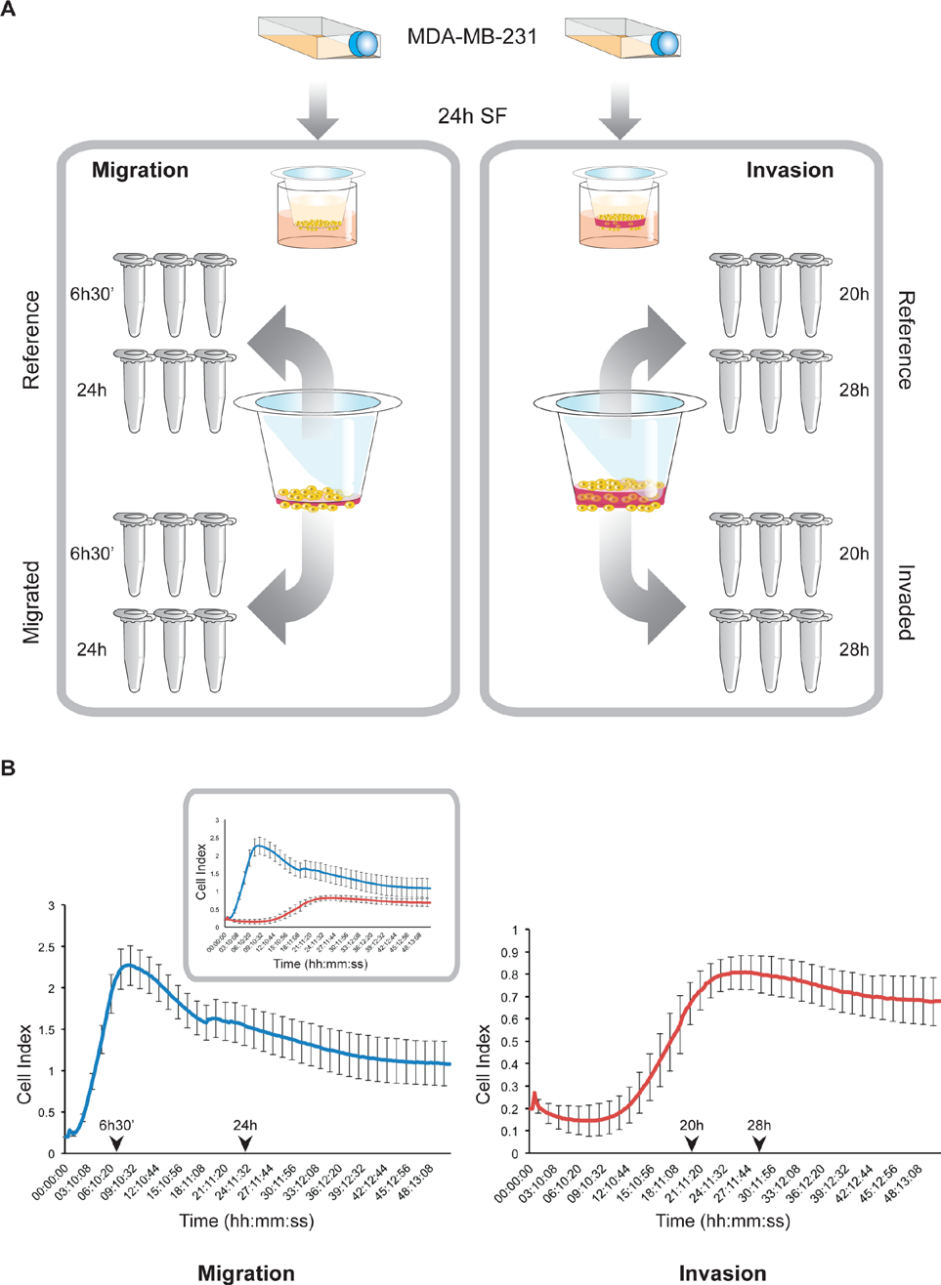
Experimental design for *in vitro* isolation of RNA from migratory/invasive and reference populations A. After 24h of serum starvation, MDA-MB-231 cells were seeded into Transwell inserts with (invasion) or without a layer of Matrigel (migration). At the indicated early and late time points, total RNA was isolated after direct lysis of the respective cell populations on the membranes. This procedure was performed in triplicate for each condition. B. Time point selection for RNA-isolation from migratory and invasive cells. Dynamic migration (left) and invasion (right) profiles of MDA-MB-231 cells have been generated by *xCELLigence* RTCA in correlating conditions with the Transwell experiments (panel A). Arrowheads indicate selected time points for RNA-isolation as described in panel A. Inset shows an integrated plot of the migration (blue graph) and invasion (red graph) patterns.

Genome-wide approaches to elucidate gene expression patterns can be applied to identify genes essentially associated with specific biological phenotypes. As cell motility and invasion have a stochastic and transient nature, in an experimental setting, migrating and invading cells need to be isolated from the general population in order to study the gene expression profiles linked to these processes [8]. This report describes microarray analysis on minute RNA-quantities derived from migrated or invaded subpopulations of triple negative MDA-MB-231 breast cancer cells in a Transwell set-up at two different time points during motility and invasion, pre-determined as “early” and “late” in real-time kinetic assessments (Fig 1). Invasion- and migration-related gene expression signatures were generated through comparison with non-invasive cells, remaining at the upper side of the Transwell membranes. Late-phase signatures of both invasion and migration indicated poor prognosis in a series of breast cancer data sets. Furthermore, evaluation of the genes constituting the prognostic invasion-related gene signature revealed a differential expression of members of the Krüppel-like transcription factor family (*KLF*s) and more specifically, Krüppel-like factor 9 (*KLF9*) was identified as a potential key player in invasive growth of breast cancer.

## RESULTS

### Expression profiling of migratory and invasive breast tumor cells

Initially, to determine the time dependent motion kinetics of MDA-MB-231 cells, real-time impedance-based recording of invasion and migration was performed, revealing different phases in both processes (Fig 1). After selection of two time points (“early” and “late”), RNA from invasive and migratory MDA-MB-231 cells was isolated from Transwell membranes and hybridized onto an Illumina HumanHT-12 v4 Expression beadchip. When comparing gene expressions of migrated vs reference cells, we identified 943 and 1622 unique and differentially expressed genes at the early and late time point respectively. For both time points, approximately half of the differentially expressed genes were upregulated in the motile cell fraction (respectively 47% and 52%). Similar analysis of the expression profiles of the invasive cells resulted in 3116 and 1060 unique and differentially expressed genes in the early and the late time points respectively. Again, for both time points, approximately half of the differentially expressed genes were upregulated in the invasive cell fraction (respectively 45% and 50%). Lists of differentially expressed genes are provided in the supplementary table S1. GSEA and IPA suggest that NFkB-signaling, cell death and attenuated cell proliferation are characteristics of early migratory cells whereas at later time points, migratory cells show evidence of active cell proliferation. Invasive cells exhibit a remarkably similar and time point-independent biological profile characterized by attenuated Interferon type 1 signaling and active DNA metabolism. Remarkably, diverse pathways of DNA-replicaton and repair, double strand break repair and damage response were found to be significantly enriched in invasive cells (supplementary figure 1). Detailed results, including the top-scoring network for each gene list identified by IPA, are provided in the supplementary table S2.

### Generation of gene signatures for migratory or invasive breast cancer cells

Using the nearest shrunken centroid-algorithm, we identified gene signatures representing molecular changes occurring either early or late during the acquisition of a motile or invasive cell phenotype. For each condition, the δ-value, the corresponding cross-validated error rate and the number of genes retained in the signatures are provided in Table 1. The genes constituting the signatures are indicated in the respective lists of differentially expressed genes (supplementary table S1). When applying the early and late invasion gene signatures onto the gene expression profiles from a collection of breast cancer cell lines classified as “invasive” or “non-invasive” [12], both signatures achieved a sensitivity and specificity of 83% and 58% respectively.

**Table 1:** 

Condition	Delta	ER-LOOCV	# genes	Test ER	Sensitivity	Specificity
Early Migration	2.1	0%	271			
Late Migration	3.1	0%	211			
Early Invasion	2.7	0%	201	37%	83%	58%
Late Invasion	2.8	0%	255	37%	83%	58%

ER Error Rate LOOCV Leave-One-Out Cross-ValidationFigure Legends

### Application of gene signatures on breast cancer expression series

To evaluate the clinical relevance of the identified gene signatures, each of them was applied onto four gene expression data sets, comprising for a total of 979 samples from patients with breast cancer. Across all signatures, about 48% (range: 47% - 49%) of the samples were predicted to exhibit migratory or invasive properties. For each signature, the percentage of samples with presumed migratory or invasive properties for each data set and their range of posterior probabilities (i.e. indication of the robustness of classification) are provided in supplementary table S3. When comparing the classifications obtained for each of the signatures, we observed significant agreements between the classification results (average OR: 6.602; range ORs: 2.601-14.241; all P<0,001) indicating that migration and invasion are related biological processes in breast cancer biology, independent of the evaluated time point. When comparing the classification results to the molecular subtypes, we observed augmented posterior probability scores for all the signatures in basal-like breast tumors and attenuated posterior probability scores in luminal A breast tumors (Kruskal-Wallis test; all P<0.001). These results were corroborated by correlation analyses, comparing the posterior probability scores obtained for each signature with PAM50-derived scores for basal-like, HER2-enriched, luminal A, luminal B and normal-like breast cancer. In addition, positive correlations were observed between the posterior probability scores and the PAM50-derived cell proliferation score, particularly for the signatures associated with the late time points. Results from the correlation analyses are provided in supplementary table S3.

**Figure 2:**
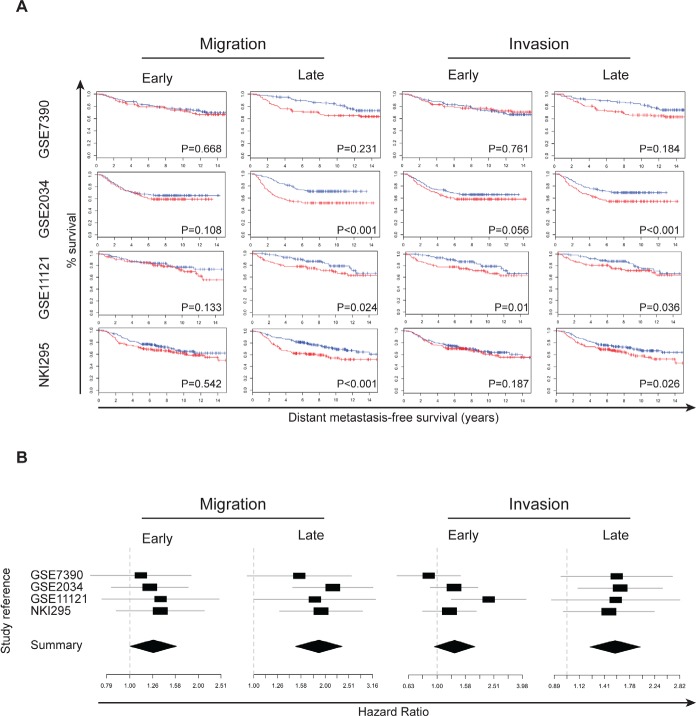
Gene signatures from migratory and invasive breast cancer cells indicate reduced DMFS A. Kaplan-Meier survival analysis for four different gene signatures on four breast cancer gene expression datasets. Columns 1 – 2 represent the early and late migration signature, column 3 – 4 the early and late invasion signature respectively. Rows 1 – 4 represent datasets GSE7390, GSE2034, GSE11121 and NKI295 respectively. Tumor samples have been dichotomized according to the degree of correlation with the respective signatures: < 50% (black graph) and > 50% (red graph). P-values are depicted on each plot. B. For the gene signatures related to early migration (top left), late migration (top right), early invasion (bottom left) and late invasion (bottom right) forest plots were generated depicting the associations of each signature with DMFS per data set. The X-axis represent the hazard ratio obtained through cox regression analysis. The black square indicates the magnitude of the hazard ratio and is proportional to the sample size in the corresponding study. The grey line indicates the 95% confidence interval. The black diamond indicates a summary value estimated through meta-analysis using a random effects model. The width of the diamond is proportional to the 95% confidence interval. The dashed vertical grey line indicates a hazard ratio of 1, meaning no association with DMFS. Confidence intervals crossing this grey line represent non-significant hazard ratios.

We next evaluated the prognostic performance of the gene signatures representing molecular changes either early or late during the acquisition of a motile or invasive cell phenotype. For each data set and each signature separately, a Kaplan-Meier analysis and univariate Cox regression analysis were performed. For the Cox regression analyses, hazard ratios, confidence intervals and P-values are provided in supplementary table S3. In addition, Kaplan-Meier plots are provided in Figure 2A and forest plots in Figure 2B. In general, patients with tumors with enhanced migratory or invasive characteristics exhibit a reduced DMFS interval, independent of the evaluated time point. Univariate Cox regression analysis was then performed on the combined data set of 979 samples. In this series we observed significantly reduced DMFS intervals for patients bearing tumors that exhibit characteristics of migratory or invasive breast cancer cells, independent of the evaluated time point. Data are provided in supplementary table S3. In multivariate analysis, after a stepwise backward selection procedure, only the PAM50-derived scores for luminal A or normal-like tumors remained significant in addition to the PAM50-derived score for cell proliferation.

### Identification of KLF9 as a potential suppressor of invasive growth in breast cancer cells

**Figure 3:**
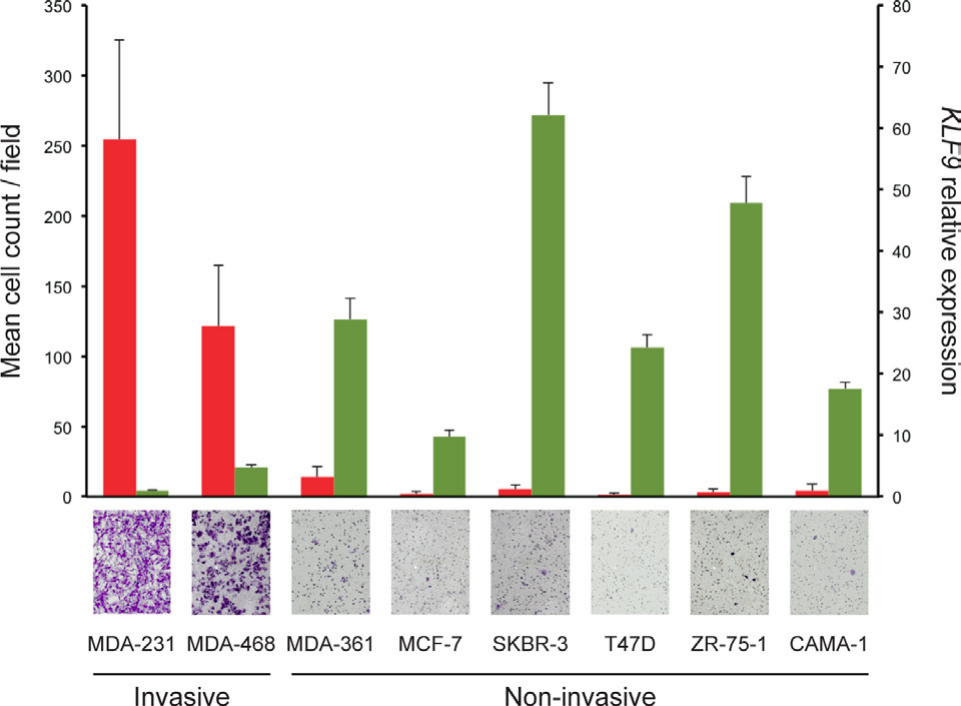
*KLF9* is downregulated in invasive cell lines Non-invasive breast cancer cell lines show higher expression levels of *KLF9* mRNA than invasive cell lines. Results from Transwell Matrigel invasion experiments are shown in red (left axis), accompanied by micrographs of representative crystal violet-stained membranes (obj. 20X) per cell line mentioned below. All experiments have been performed in technical and biological triplicates. Normalized relative expression levels for *KLF9* as measured by RT-qPCR are shown in green (right axis). All RT-qPCR experiments have been performed in at least technical duplicates and biological triplicates. All results shown are means + SD.

The microarray-based gene expression studies revealed that *KLF9* (also known as BTEB1) was significantly downregulated in the invasive subpopulation of MDA-MB-231 cells. This finding has been validated by RT-qPCR (Supplementary Fig 2) and possibly implies a suppressing capacity on breast cancer cell invasiveness. Therefore, we screened a panel of breast cancer cell lines for *KLF9*-expression using RT-qPCR. It was found that non-invasive cell lines (MCF-7, SKBR-3, T47D, ZR-75-1, CAMA-1 and MDA-MB-361) showed significantly higher levels of *KLF9* mRNA when compared to invasive cell lines (MDA-MB-231 and MDA-MB-468) (Fig 3).

**Figure 4:**
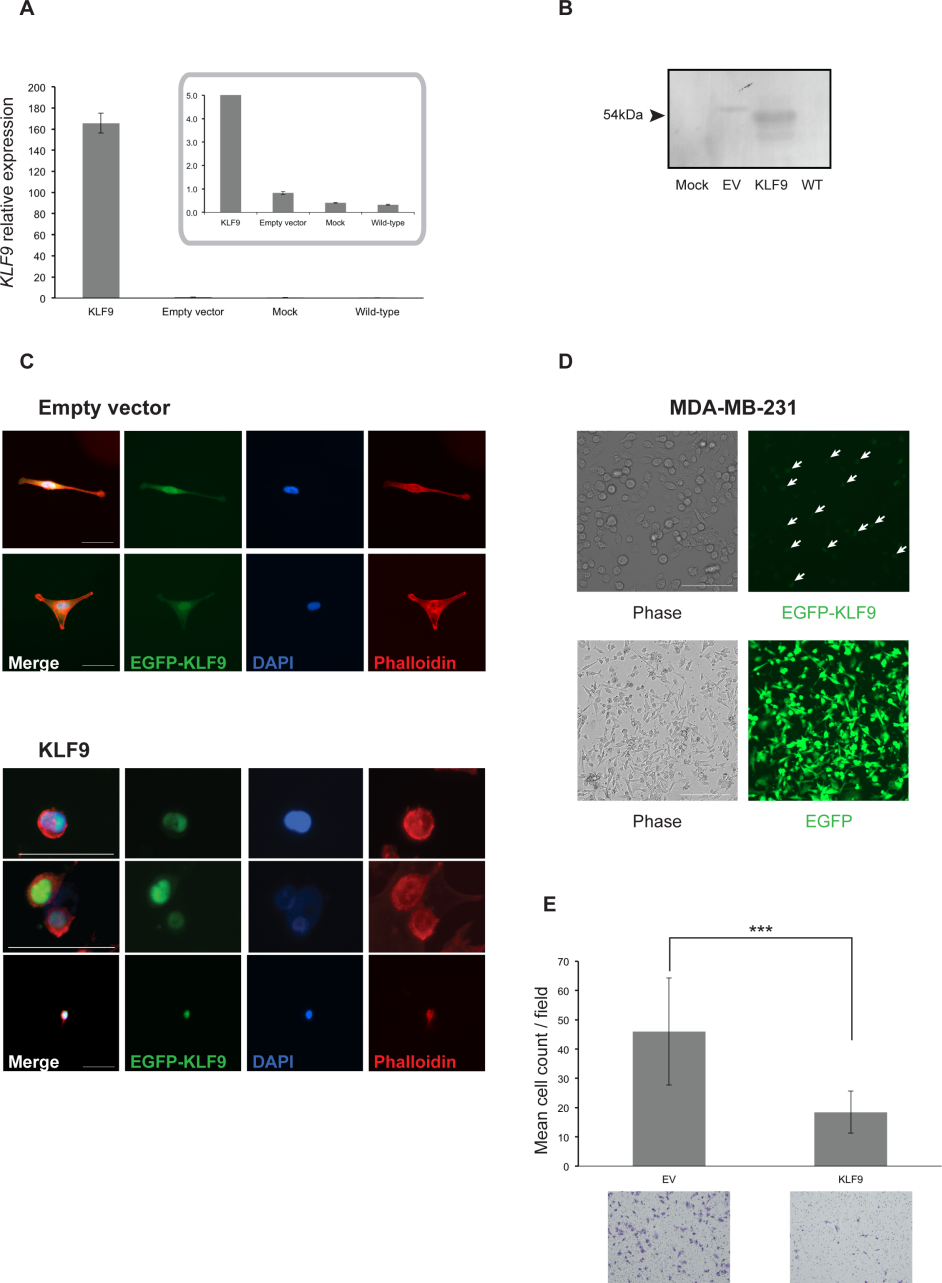
Forced expression of *KLF9* alters cell morphology and impairs breast cancer cell invasion **A.** Normalized relative expression levels of *KLF9* in transiently transfected MDA-MB-231/*KLF9* and MDA-MB-231/EV cells 24h post-transfection. Inset shows detailed results for MDA-MB-231/EV, mock-transfected and wild-type MDA-MB-231. Results shown are means ± SD of three replicates. **B.** Western blot analysis for expression of EGFP-KLF9 fusion protein in MDA-MB-231 cells, transfected with either *KLF9* or EV. Lysates were prepared 24h after transfection. **C.** F-actin staining of MDA-MB-231/*KLF9* and EV using phalloidin-TRITC. EV-transfected cells reconstitute the spindle-like morphology of wild-type MDA-MB-231 cells. EV shows both nuclear and cytosolic localization (EGFP, green). Phalloidin-stained actin (red) indicates changes in cell morphology in *KLF9*- and EV-expressing cells. Bars: 50μm. **D.** Phase contrast (left column) and fluorescence images (right column) of MDA-MB-231/*KLF9* (top row) and EV (bottom row) respectively. Pictures were taken after 16 days of culturing in the presence of G418 selection antibiotic for enrichment of transfected cells. KLF9-expressing MDA-MB-231 cells (top row) are incapable of reconstituting the wild-type morphology. Arrows indicate examples of cells expressing *KLF9*. Bars: 100μm (top row) and 200μm (bottom row). **E.** Transwell-based Matrigel invasion experiments revealed impairment of MDA-MB-231 invasion in the presence of KLF9. Results shown are mean cell counts ± SD from three independent experiments in at least experimental triplicates. *** P < 0.001 (Mann-Whitney U)

To better address its role in breast cancer cell motility and invasion, *KLF9* was overexpressed in MDA-MB-231 cells by transient introduction of a pEGFP-*KLF9* fusion construct and a pEGFP plasmid in parallel serving as control. Expression was confirmed by both RT-qPCR, immunofluorescence and Western blot (Fig 4A, B). In addition, it must be noted that expression of the EGFP-KLF9 fusion protein was strictly confined to the nucleus whereas EGFP only (EV) was localized in nuclear and cytosolic areas (Fig 4C), indicating intact functionality of the fusion protein.

Introduction of EGFP-KLF9 induced a morphological switch in MDA-MB-231 cells after 24h, leading to reduced spreading and the formation of dense zones of cortical actin (Fig 4C). As shown, MDA-MB-231/EGFP cells maintained the wild-type spindle-shaped morphology. This finding has been confirmed after 16 days of culturing in complete growth medium containing G418 selection antibiotic hereby enriching for MDA-MB-231/EGFP-KLF9 resp. MDA-MB-231/EGFP cell populations (Fig 4D).

Putative involvement of *KLF9* in breast cancer cell invasion was assessed *in vitro* by conducting a series of Transwell experiments on transiently transfected MDA-MB-231 cells after 24h. MDA-MB-231/EGFP-KLF9 cells showed a significantly decreased invasive potential when compared to MDA-MB-231/EGFP (P < 0.001, Mann-Whitney U) (Fig 4E).

### *KLF9* is downregulated in breast tumor tissue

**Figure 5:**
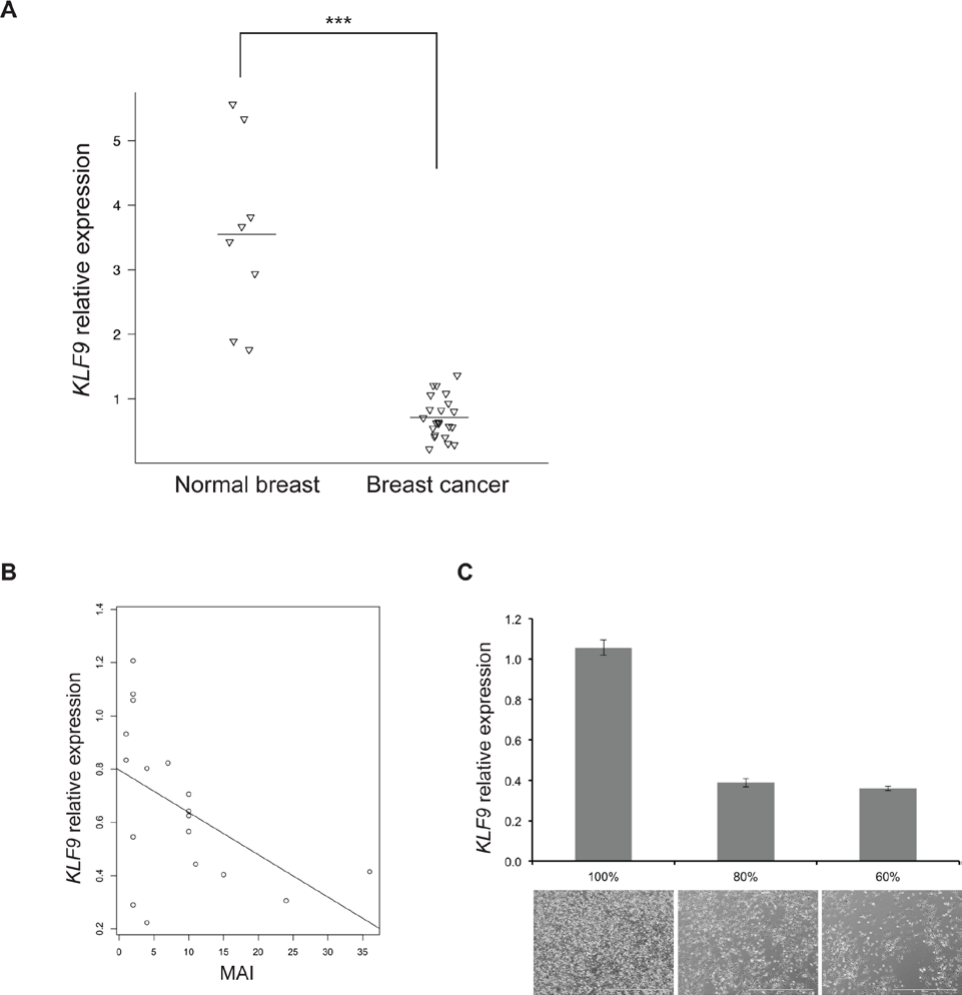
*KLF9* is downregulated in human breast cancer **A.** Scatter plot depicting normalized relative expression levels of *KLF9* as detected by RT-qPCR in normal human breast samples (N=8) and breast cancer (N=22). Horizontal lines indicate the mean level of expression per group. *** P < 0.001. **B.** Correlation plot showing available mitotic activity indices (MAI), as assessed at diagnosis for each patient (N=18), in relationship to the normalized expression of *KLF9* per sample. **C.** Normalized relative expression levels of *KLF9*, as detected by RT-qPCR in triplicates, in wild-type MDA-MB-231 cells at different degrees of confluency in culture, illustrated by phase contrast pictures below each degree. Bars: 1000μm.

To investigate the clinical relevance of the observed *in vitro* effects, the expression of *KLF9* was assessed in normal human breast tissue and breast cancer. All breast cancer samples (N=22, mean age: 59 ± 13) showed a significantly decreased expression of *KLF9* in comparison with normal breast tissue (N=8, mean age: 45 ± 16) (Fig 5A).

Oncomine (www.oncomine.com, Oncomine Research Premium Edition) [13] was searched for *KLF9* in several expression data sets containing mixed normal and cancer samples. Next to loss of expression in 6 out of 16 cancer types (Supplementary figure 3A), *KLF9* was shown to be significantly downregulated in breast carcinoma when compared to normal breast epithelium, applying P ≤ 0.01 and fold change ≥ 2 as thresholds (Supplementary figure 3B-D, Supplementary table S4 ) ([14, 15] and TCGA Research Network). This was also found in data sets comparing invasive breast carcinoma with normal breast tissue (Supplementary figure 3E-F, Supplementary table S4) ([16] and TCGA Research Network). There was no correlation of *KLF9*-expression with ER-, PR- or HER2-status, implying that loss of expression of this transcription factor is a receptor-independent event in the malignant transformation of mammary epithelial cells.

Additionally, an inverse correlation was found between *KLF9*-expression and the Mitotic Activity Index (MAI) in the series of breast cancer samples (Fig 5B), implying an anti-proliferative effect of *KLF9*. *In vitro*, upregulation of *KLF9* expression was shown in MDA-MB-231 cells in a confluency-dependent manner with limited expression in subconfluent cultures (60% - 80%) and a 3-fold rise of *KLF9* mRNA in confluent cultures, corroborating the findings in clinical samples (Fig 5C).

## DISCUSSION

This report describes the assessment of gene expression characteristics associated with breast cancer cells in two different phases during migration and invasion. It has become clear that a high degree of heterogeneity exists both between (*inter-*tumoral) and within (*intra-*tumoral) individual breast tumors [17]. It has already been established that, within a clinically invasive tumor, not all tumor cells will become invasive and that invasive behavior is regulated spatio-temporally by the microenvironment [10]. The molecular characterization of cancer cell migration and invasion by genome-wide gene expression profiling is largely impeded by these features. When obtaining microarray-based profiles of cells exposed to chemotactic cues, the expression profile of the motile / invasive subpopulation of interest is masked by non-responsive cells constituting the majority of the cell population [6]. The triple negative MDA-MB-231 cell line is able to spontaneously metastasize in orthotopic mice models and, therefore, represents a suitable model to detect gene expression changes associated with the early steps of metastatic dissemination [18]. In the present study, a Transwell-based design was adopted in order to capture the gene expression characteristics of the migrated/invaded subpopulation, physically separated from the non-migratory / non-invasive majority of cells. Furthermore, two time points have been selected to distinguish gene expression patterns in an early and a late phase and invasion and migration have been regarded as distinct processes *in vitro*. Differential gene expression and pathway analysis revealed that the invaded and migrated subpopulations were, next to processes of cell movement, highly enriched in replicative, inflammatory and developmental programs. In addition, it was found that both invaded and migrated subpopulations showed significantly upregulated DNA damage repair activity (supplementary fig 1). These findings correlate with results of a recent study with a similar rationale, conducted *in vivo* using the same cell line [18]. Moreover, in concordance with Patsialou et al., invaded cells showed increased TGFß-signaling in the early phase, without significant detection of TGFß itself and upregulation of *CDC25A*, a gene associated with tumorigenesis [19].

The gene expression signatures, corresponding with migrated and invaded cells in the late phase, consequently predicted reduced DMFS for the four expression datasets, comprising in total nearby 1000 breast tumors. The positive correlations with the PAM50-derived cell proliferation score obtained for the late-phase signatures, however, indicate proliferation to remain a driver in the prognostic ability, even though motility and invasion were the experimental foci in this study.

Nevertheless, the identification of *KLF9* and the initial results showing its inhibitory effect on cancer cell invasion, corroborated by its differential expression between invasive and non-invasive cell lines, imply interactions between proliferation- and invasion-mediating programs. *KLF*s form a family of highly conserved zinc finger transcription factors with versatile roles in proliferation, differentiation and development [20]. Apart from *KLF9*, our data also demonstrate downregulation of *KLF4* and *KLF6* in the early invaded and late migrated subpopulations respectively. To date, *KLF4* is the most extensively described member of the family, not in the least for its contribution to pluripotent stem cell induction [21]. In cancer biology, the effect of *KLF*s has been found to be context-dependent, with both tumor suppressing and oncogenic roles reported for breast cancer [22-24]. This duality could, at least partly, be explained through altered genetic backgrounds appearing in transforming epithelial cells [25]. Altogether, the most recent reports point towards *KLF4* as a tumor suppressor, being downregulated in human breast cancer samples. Additionally, a promoting interaction between *KLF4* and *CDH1* (E-cadherin) has been described, hereby driving cancer cells into an EMT-program through loss of *KLF4* [23]. A similar effect was found to be exerted through downregulation of *KLF17* [26]. *KLF6* has been reported as a tumor suppressor in prostate cancer [27] and recently a splice variant thereof, KLF6-SV1, was identified as an oncogene in breast cancer with increased expression in breast tumors [28]. Our findings support these results, showing loss of expression of these *KLF*s in migratory / invasive breast cancer cells, and illustrate the complexity of their role in tumor development and progression. Reports on the role of *KLF9* are thus far limited to colon and endometrial carcinoma. Reduced expression of *KLF9* has been shown on a transcriptional as well as protein level in colorectal cancer samples when compared to paired normal mucosa [29]. Similarly, endometrial carcinomas also demonstrated lower levels of *KLF9* when normal endometrium and stage I disease were compared with stage II – IV carcinomas. In this tumor type, downstream repression of regulators of the actin cytoskeleton has been detected by microarray in modified cell lines overexpressing *KLF9* [30]. Our results provide the first evidence of *KLF9* as a suppressor of invasive growth in breast carcinoma. The loss of *KLF9,* as found in several Oncomine breast data sets and in a series of breast samples from our institution, confirm the clinical relevance of this transcription factor in human breast cancer. The inverse relationship of *KLF9* with mitotic activity and the contact inhibition-driven upregulation of *KLF9* in culture, imply a role in cell cycle regulation, which has also been demonstrated for *KLF4* [31]. Whether the reduced expression of *KLF9* in invaded MDA-MB-231 cells in comparison with their non-invasive counterparts is an effect of active transcriptional or translational repression or due to the presence of a “*KLF9*_low_”-subpopulation within the general cell population remains to be investigated.

Our data set derived from invaded breast cancer cells also revealed a significant downregulation of E74-like factor 3 (*ELF3*). *ELF5*, a transcriptional regulator closely related to *ELF3*, has recently been identified as a suppressor of EMT in MDA-MB-231 cells by direct repression of *SNAI2*. In addition, *ELF5*-expression was strongly reduced in breast cancer samples [32]. Also recently, leukemia inhibitory factor receptor (*LIFR*), a gene present in our prognostic invasion signature, was found to exert anti-metastatic effects through activation of the Hippo pathway and functional inactivation of the transcriptional co-activator *YAP*. A significant association between loss of *LIFR* and poor prognosis in breast cancer has been discovered as well [33]. Indeed, our data from isolated invaded MDA-MB-231 cells reveal a significant downregulation of *LIFR*, in support of these recent results.

In conclusion, our data provide novel insights on cell migration and invasion as separate and initiating processes in the invasion-metastasis cascade. The devised *in vitro* experimental design has yielded prognostic gene signatures indicating reduced DMFS, identified *KLF9* as a novel potential invasive growth suppressor in breast cancer and generated results in concordance with previous studies. Gene expression profiling has indeed revealed migration and invasion as highly related, yet different processes. Furthermore, according to our results, the role of proliferation, DNA damage repair mechanisms and cell motility programs should not be considered as mutually exclusive. We believe that the consistent subtype-independent downregulation of *KLF9* in patient material confirms the relevance of this factor in human breast cancer. Finally, the complex interplay of *KLF9* with its related family members in breast cancer, and epithelial cancer biology in general, still remains largely elusive and needs to be thoroughly investigated in order to identify downstream genes as potential therapeutic targets or mediators of metastatic progression.

## Materials and methods

### Cell culture

The MDA-MB-231, MCF-7, CAMA-1 and ZR-75-1 cells were cultured in RPMI1640, MDA-MB-468 cells in DMEM/F12, MDA-MB-361 and T47D cells in DMEM and SKBR-3 cells in McCoy's 5A media, all supplemented with 10% fetal bovine serum (FBS), 1% L-glutamine, 1% penicillin/streptomycin and 1% sodium pyruvate. All cell culture reagents were purchased from Invitrogen (Life Technologies, USA) unless mentioned otherwise. Cell lines were maintained at 37°C and 5% CO_2_/95% air in a humidified incubator. MDA-MB-361 and CAMA-1 were kindly provided by Dr. Maurice PHM Jansen and Dr. John WM Martens (Department of Medical Oncology, Erasmus University Medical Center, Daniel den Hoed Cancer Center, Rotterdam, The Netherlands). Although the MDA-MB-231, MDA-MB-468, MCF-7 and SKBR-3 breast cancer cell lines have been purchased from the American Type Culture Collection (ATCC, USA) (http://www.lgcstandards-atcc.org), all cell lines have been validated in-house by short tandem repeat (STR) profiling using the Cell ID™ System (Promega, USA) according to the manufacturer's instructions.

### *xCELLigence* real-time cell analysis (RTCA)

Real-time cell migration and invasion experiments were performed on an *xCELLigence* RTCA DP instrument (ACEA Biosciences, USA) as described previously [34]. Briefly, 1.6x10^5^ MDA-MB-231 cells were seeded on top of a solidified Matrigel layer (10% v/v) in kinetic correlation with 4x10^5^ cells on 20% (v/v) Matrigel in a conventional Transwell system [34]. Cells were allowed to invade during 50h with kinetic measurements programmed every 15 minutes to display dynamic invasive behavior patterns at the above mentioned cell and Matrigel densities. Cell migration experiments were carried out as described above, without application of Matrigel.

### Transwell invasion and migration assay

#### Migration and invasion set-up for RNA-isolation of distinct cell populations

*In vitro* invasion experiments have been performed using a conventional 24-well Transwell system (Corning®), essentially as described previously [34], with 4x10^5^ cells seeded on top of a 20% (v/v) Matrigel layer. Complete medium was added to the wells as chemoattractant and the Transwell plates were incubated at 37°C/5% CO_2_ during 20h (first timewindow) and 28h (second timewindow), as predetermined in real-time invasion assessments.

An identical protocol as the above was followed for migration experiments, without application of an extracellular matrix substitute. Transwell migration plates were incubated during 6h30' (first timewindow) and 24h (second timewindow). For both invasion and migration, the respective timeframes of incubation have immediately been followed by RNA-isolation.

#### Migration and invasion assessment of wild-type and transfected cell lines

Cell line Transwell migration and invasion experiments were performed essentially as described above with the following specifications for transfected cell lines: 24h after transfection, 10^5^ cells were seeded in the inserts after 4h serum starvation. Both invasion and migration were quantified after 24h by cell counting in at least three different fields per membrane at 10X-magnification. Each condition was performed in technical and biological triplicates.

### RNA-isolation from invasive and migratory subpopulations and microarray hybridization

Total RNA was extracted using the RNAqueous™ Micro Kit (Life Technologies, USA) for nucleic acid extraction from small cell populations. All cell populations of interest were lysed directly on the membranes without prior enzymatic detachment. Isolated RNA was immediately tested on yield, purity and quality using a NanoDrop® ND-1000 (Thermo Scientific, USA) and a BioAnalyzer 2100 (Agilent Technologies, USA) device. Samples were stored at -80°C and only samples with absorption (A) ratios A_260_/A_280_ ≥ 1.8 and A_260_/A_230_ ≥ 1.5 (NanoDrop®) and RNA Integrity Number (RIN) > 6 (BioAnalyzer) were considered for microarray hybridization.

Prior to hybridization, RNA samples were amplified using the Illumina Totalprep RNA Amplification kit (Life Technologies, USA). Subsequently, single-stranded cRNA with incorporated biotin-UTP nucleotides was produced by an *in vitro* transcription reaction and hybridized onto an Illumina Human HT-12 v4 gene expression BeadChip. Three biological replicates per time point per biological state were loaded, totalling 24 sample hybridizations on two chips. After overnight sample hybridization, subsequent washing steps and sample labeling, intensity values were read on an Illumina iScan equipped with iScan control software (v. 3.3.29). All microarray expression data have been deposited in the Gene Expression Omnibus database (http://www.ncbi.nlm.nih.gov/geo/) under the accession number GSE54465.

### RNA-isolation from cell lines and clinical samples

Cell line total RNA was extracted using the TRIzol® method (Life Technologies, USA) after lysis of subconfluent cultures in 25cm^2^ culture flasks (T25). As mentioned above, yield and purity of the isolates were tested using a NanoDrop® system and stored at −80°C.

From every clinical sample, 6-12 10μm-sections were made and stored in cryotubes (−80°C) for RNA-extraction. RNA was extracted from the samples using the RNeasy Lipid Tissue Mini Kit (Qiagen, Germany).

### Microarray data analysis

All microarray data analysis procedures were performed using BioConductor in R (v 2.13.0). Raw intensity reading, log2-transformation, summarization, quantile normalization and quality controls were performed using the methods implemented in the BioConductor package “beadarray” (v 2.8.1) [35]. Normalized expression data were further analysed using the package “limma” (v 3.14.4) [36] to identify lists of differentially expressed genes between the migratory or invasive cell populations on the one hand and their respective controls on the other hand. Lists of differentially expressed genes were then functionally annotated by analyzing them for enriched Gene Ontology (GO) and Kyoto Encyclopedia of Genes and Genomes (KEGG) categories through hypergeometric Gene Set Enrichment Analysis (GSEA). In addition, Ingenuity Pathway Analysis (IPA) software was used for similar purposes. For all analyses, correction for multiple testing was performed using the Benjamini and Hochberg step-up false discovery rate (FDR) controlling procedure and adjusted P-values inferior to 0.1 were considered significant.

To identify gene signatures (N=4) representing molecular changes occurring either early or late during the acquisition of a motile or invasive cell phenotype, the nearest shrunken centroid-algorithm, implemented in the BioConductor-package Prediction Analysis of Microarrays for R (PAMR), was applied. Using Leave-One-Out Cross-validation (LOOCV), a δ-value that corresponds to the lowest cross-validated error rate, was selected. This δ-value defines a list of predictor genes for which the minimal expression difference between the invasive or motile cell populations and their controls equals δ. The thus identified lists of predictor genes determine centroids that can be used to classify patient samples according to the presence or absence of motile or invasive cancer cell characteristics.

To determine the clinical relevance of our gene signatures, we analyzed four publicly available gene expression series from patients with breast cancer with documented follow-up in terms of distant metastasis-free survival (DMFS; GSE2034 [37], GSE7390 [38], GSE11121 [39] and NKI295 [40]). To ensure proper classification performance, we used the “limma” package to normalize each data set. Sample classification was then performed by comparing the signature-specific expression profiles of each sample with each of the identified centroids. In this process, each sample is assigned a posterior class probability, a value ranging from 0 to 1 indicating the strength of the classification. In addition, each sample in these patient series was classified according to the molecular subtypes using the PAM50-algorithm [41].

### Survival analysis

To evaluate the prognostic performance of the gene signatures, we performed Kaplan-Meier and Cox regression analysis using DMFS as endpoint. All analyses were performed using the R-package “survival” (v 2.36.5). Kaplan-Meier analysis was performed using the dichotomized classification results (i.e. invasive/non-invasive or motile/non-motile). Univariate Cox regression analysis was performed using the posterior class probabilities for each of the identified gene signatures and all parameters (i.e. molecular subtypes and the measures for cell proliferation, ER-activation and ErbB2-activation) provided by the PAM50-algorithm. Multivariate Cox regression analysis was then performed for each variable identified as significant in the univariate setting.

### Clinical samples

The clinical breast tumor and reference normal tissue samples used in this publication were provided by the UZA tumor bank, Antwerp University Hospital, Belgium. The samples were stored at -80°C. In each case one 8μm-section was made, using a cryotome (Thermo Scientific, USA), which was HE stained (Thermo Scientific, USA). All sample diagnoses were validated by a trained pathologist, considering only samples without necrosis and, for tumor samples, a tumor/stroma ration of minimum 40%. The Mitotic Activity Index (MAI) was calculated as the average number of mitotic spindles per mm^2^, counted in 10 different high-power fields.

### cDNA synthesis and real-time quantitative PCR

#### Microarray validation

Reverse transcription quantitative PCR (RT-qPCR) was performed on total RNA in a single reaction using the Power SYBR Green RNA-To-C_T_ 1-Step kit (Life Technologies, USA) on a LightCycler 480 instrument (Roche Applied Science, Germany). Primers were designed using QuantPrime software [42] and RTPrimerDB (http://www.rtprimerdb.org) [43] and have been obtained from Integrated DNA Technologies (USA) (supplementary table S5). All reactions have been performed in triplicates in 384-well plates with 2μL RNA (prediluted to 15ng/μL) as input in a total reaction volume of 10μL, further comprising 5μL Power SYBR Green RT-PCR Mix (2x) (Life Technologies, USA), 0.08μL RT Enzyme Mix (125x) (Life Technologies, USA) and 200nM of each primer (final concentration). Normalized relative gene expression values were calculated using qBase^PLUS^ software version 1.5 (Biogazelle) [44].

#### Cell line expression levels of KLF9

All reactions have been performed following the above described protocol using identical primer pairs. *SDHA*, *RPL13A* and *HMBS* were identified as stably expressed across all analyzed cell lines and have been used as reference genes to determine relative *KLF9* expression levels in triplicate.

#### Expression levels of KLF9 in breast tumor tissue

All RT-qPCR experiments were performed as indicated above. *SF3A1* and *YWHAZ* were identified as being stably expressed in tumor as well as normal tissue samples. Expression levels of *KLF9* were calculated using qBase^PLUS^ v1.5.

### Molecular cloning and transfection

The ORF of WT *KLF9* was cloned in a pEGFP-N1 backbone, with subsequent bi-directional sequencing on an ABI 3130xL (Life Technologies, USA). Next, expression vectors were electroporated into MDA-MB-231 cells using a Nucleofector II device (Lonza, Switzerland). Briefly, 10^6^ cells were collected from a subconfluent (80%) culture, electroporated with plasmid DNA and seeded into 6-well plates. After overnight settlement, growth medium was replaced and experiments initiated after 24h.

To obtain stable cell populations Enrichment for EGFP-KLF9 or EGFP expressing cells was obtained through addition of G418 antibiotic (800μg/mL, Life Technologies, USA) to the growth medium starting 24h after transfection and renewed every 48h.

### Fluorescence microscopy

Cells were fixed in 4% paraformaldehyde, permeabilized in 0.1% Triton-X-100, blocked in 1% BSA, stained with phalloidin-TRITC (Sigma Aldrich, USA) and mounted onto glass slides using Vectashield® HardSet™ Mounting Medium (Vector Laboratories, USA) containing DAPI as nuclear counterstain. Visualization and image capturing have been performed on an EVOS® FL Cell Imaging System (Life Technologies, USA).

### Western blot

Whole-cell lysates have been prepared using RIPA buffer containing protease-inhibitors (Roche Diagnostics GmbH, Germany). Protein concentration was determined using the BCA-200 protein assay kit (Thermo Scientific, Wilmington, DE, USA). Twenty μg of proteins were loaded and separated by SDS-PAGE (Life Technologies, USA) on a 12% gel. Blots were incubated overnight with primary antibodies against KLF9 (Santa Cruz Biotechnology, USA) or EGFP (Sigma-Aldrich, USA). After incubation with a secondary IgG-HRP antibody (Santa Cruz Biotechnology, USA) and addition of HRP-substrate (Lonza, Switzerland), proteins were visualized by chemiluminiscence.

### Statistical analysis

All statistical analyses, except those related to microarray-based gene expression analyses, were performed in SPSS 21.0. A P-value below 0.05 was considered to be statistically significant. * P<0.05, ** P<0.01, *** P<0.001

Detailed methods and associated references are available in the Supplementary Information (Supplementary methods).
